# A supervised diagnostic experiment of resistance variable multifault locations in a mine ventilation system

**DOI:** 10.1038/s41598-023-32530-7

**Published:** 2023-03-31

**Authors:** Dong Wang, Jian Liu, Deng Lijun, Wang Honglin

**Affiliations:** 1 College of Safety Science and Engineering, Liaoning Technical University, Huludao, 125105 Liaoning China; 2 Key Laboratory of Mine Thermo-Motive Disaster and Prevention, Ministry of Education, Huludao, 125105 Liaoning China

**Keywords:** Environmental sciences, Energy science and technology, Engineering

## Abstract

The diagnosis of resistance variable multifault location (RVMFL) in a mine ventilation system is an essential function of the mine intelligent ventilation system, which is of great significance to mine-safe production. In this paper, a supervised machine learning model based on a decision tree (DT), multilayer perceptron (MLP), and ranking support vector machine (Rank-SVM) is proposed for RVMFL diagnosis in a mine ventilation system. The feasibility of the method and the predictive performance and generalization ability of the model were verified using a tenfold cross-validation of a multifault sample set of a 10-branch T-shaped angle-joint ventilation network and a 54-branch experimental ventilation network. The reliability of the model was further verified by diagnosing the RVMFL of the experimental ventilation system. The results show that the three models, DT, MLP, and Rank-SVM, can be used for the diagnosis of RVMFL in mine ventilation systems, and the prediction performance and generalization ability of the MLP and DT models perform better than the Rank-SVM model. In the diagnosis of multifault locations of the experimental ventilation system, the diagnostic accuracy of the MLP model reached 100% and that of the DT model was 44.44%. The results confirm the MLP model outperforms the three models and can meet engineering needs.

## Introduction

The main function of the mine ventilation system is to provide fresh air to underground places that need wind. This dilutes and removes toxic and harmful gases, such as gas, carbon monoxide, and dust. It can also create a good working environment to ensure the occupational health of workers and the normal conduct of production activities^1–3^. A good ventilation system can effectively reduce the possibility of accidents, such as gas or coal dust combustion and explosion, carbon monoxide poisoning, and asphyxiation, in mines^4,5^. This shows that a stable and reliable ventilation system is extremely important for ensuring the mine’s safe production. However, during the production process of a mine, sudden changes in the air volume of the ventilation system inevitably occur, such as the blockage of the roadway bubble fall, breakage and failure of dampers, and emptying of the mine silo. The essence of these phenomena, which result in sudden changes in roadway air volume, is the sudden change in the wind resistance of the roadway. In this case, these phenomena are defined as the occurrence of resistance failure in the mine ventilation system^6^. When a resistance fault occurs in a mine ventilation system, the air volume distribution in the ventilation system changes significantly. This most likely leads to a decrease in the air supply in the mining and digging working faces, as well as the accumulation of toxic and harmful gases in some breeze tunnels. It will cause serious safety hazards and risks to the mine^7^.

The mine ventilation network has good self-adaptability and robustness, making it suitable for the application of artificial intelligence and machine learning methods^8^. Owing to the rapid development of intelligent technology, the traditional method of relying on personnel to identify resistance variable faults in ventilation systems has gradually been replaced by intelligent diagnostic methods. The intelligent diagnosis method can save considerable human and material resources. Additionally, it saves a lot of time and adapts to the demand for the rapid disposal of mine ventilation system faults. Studies have shown that artificial intelligence and machine learning algorithms, such as support vector machine (SVM), decision tree (DT), artificial neural network (ANN), random forest (RF), genetic algorithm (GA), and multilayer perceptron (MLP), are used to solve single fault diagnosis problems in mine ventilation systems^9–14^. However, owing to the specificity and complexity of underground mine conditions, it is common for mine ventilation systems to have resistance variable faults in multiple locations concurrently. Few studies have been conducted on diagnosing and identifying faults in multiple locations of mine ventilation systems.

Wang et al.^15^ first proposed a machine learning–k-nearest neighbor-based (ML–KNN-based) model and method for the diagnosis of resistance variable multifault location (RVMFL) in mine ventilation systems. They solve the RVMFL diagnosis problem of mine ventilation systems as a multilabel and multi-classification problem. From the multifault location diagnosis problem, the multilabel classification problem can be transformed into multiple single-label classification problems through a conversion strategy. For example, the ventilation system RVMFL diagnosis problem can be divided into multiple single-fault location diagnosis problems, but this undoubtedly increases the computational complexity^16,17^. The multilabel classification problem can also be solved by applying multilabel classification support and adaptation algorithms, such as DT, MLP, ranking support vector machine (Rank-SVM), and AdaBoost.MH, ML–KNN^18–22^. These methods are all supervised machine learning algorithms, and Rank-SVM is an improvement of the SVM algorithm. According to the existing studies, DT, MLP, and SVM perform well in solving the problem of resistance variable single-fault location diagnosis in the mine ventilation system^9^. In addition, these methods represent an important value in the application of classification problems. Formally due to their good classification performance and adaptation to multi-label classification problems, the methods for intelligent diagnosis of RVMFL in ventilation systems are improved. In this paper, three important and widely used machine learning algorithms, DT, MLP, and Rank-SVM, are used to investigate the problem of diagnosing RVMFL in mine ventilation systems.

It is well known that different algorithms exhibit different prediction accuracies, performances, and generalization capabilities. Industrial tests are frequently conducted to confirm whether these algorithms can be used in engineering practice, how well they perform in RVMFL diagnosis, and how reliable and valid each diagnosis model is. To conduct an industrial field test of a ventilation system failure in a production mine, it is necessary to open the dampers in a closed state for a long time or block the tunnel to make a real failure in the mine, particularly to create a failure in multiple locations. However, such a test is not permitted, particularly in coal mines. If the resistance variable fault industrial test is conducted in metal mines, unlike coal mines, metal mines do not experience gas disasters and natural coal fires in the mining area while collecting test sample data. It may lead the mine ventilation system to a state of failure for a long time, which significantly affects the safety production of mines. In summary, conducting industrial tests on actual resistance variable faults in mine ventilation systems is difficult and involves certain safety risks. To address the problem of creating resistance variable faults in actual mines that affects normal production, a ventilation system resistance variable fault simulation experimental system was built. The advantage of this system is that it can simulate any type of resistance variable faults and create any degree of resistance variable faults at any location, without being limited by the site's environmental conditions and without safety problems, such as industrial tests in the field.

**Figure 1:**
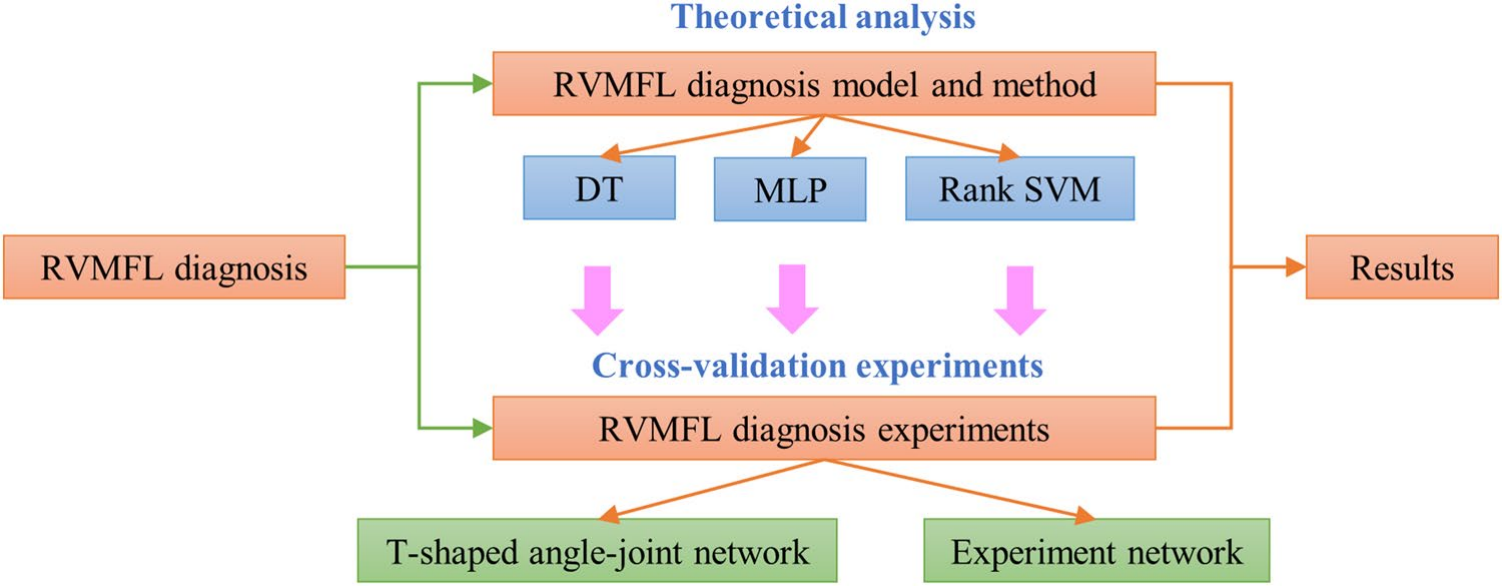
Research flowchart.

The main objectives of this study are as follows: (1) Solving the problem of accurate diagnosis of resistance variable faults occurring at multiple locations in the mine ventilation system simultaneously. (2) Analyzing and comparing the performance of different intelligent algorithms in the problem of multifault location diagnosis of resistance variable faults in ventilation systems to find more suitable algorithms and models. (3) Building an experimental system for simulating resistance variable faults in the mine ventilation system. Then, using this system to verify the reliability and validity of the proposed model through experiments, and solve the problem of being unable to conduct realistic industrial tests to confirm the reliability and validity of the algorithms in actual production mines. (4) The study results provide a theoretical basis for constructing an intelligent body on mine ventilation systems. The research flow of this paper is shown in Fig. 1.

## Method and model for RVMFL diagnosis in a mine ventilation system

### Diagnosis method for RVMFL in the mine ventilation system

**Figure 2:**
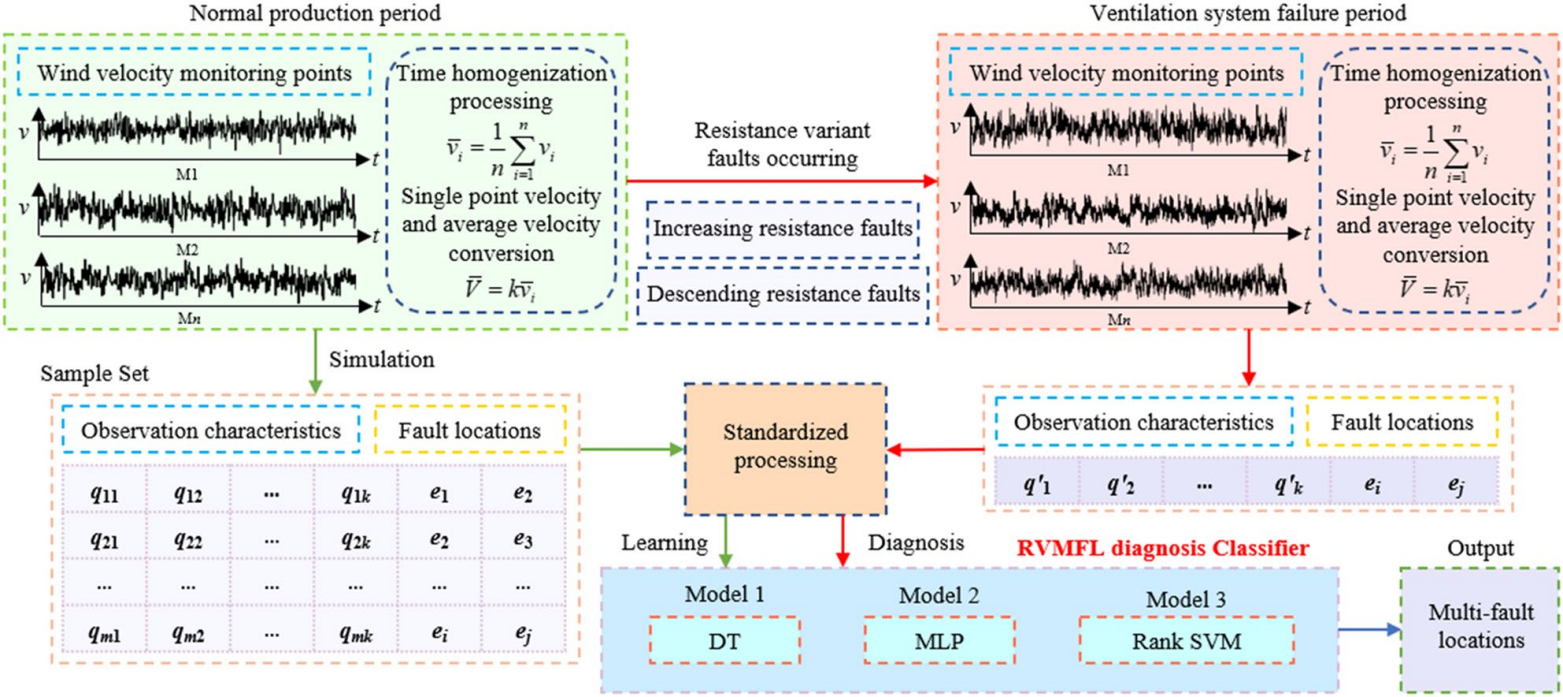
Ventilation system RVMFL diagnosis method process.

Mine occurs resistance variable multifaults refer to cases in which the mine ventilation system in a normal production period experiences resistance variable faults in two or more different roadways simultaneously. This study considers the case of two roadways simultaneously occurring with resistance variable faults as an example and adopts a supervised learning method to address the problem of mine ventilation system RVMFL diagnosis. Figure 2 shows the flow of the diagnosis method for the RVMFL of the mine ventilation system. The essence of the RVMFL diagnosis of a mine ventilation system is to construct a resistance variable fault multilabel classifier. This classifier can quickly diagnose and identify the locations of resistance variable multifaults based on the airflow information of the ventilation system after the faults occur.

The airflow information of all or the residual branches of the ventilation network is used as an observation feature. Based on the values monitored by the mine wind speed sensors, the monitored values are averaged statistically for time averaging, corrected for single-point wind speed values, and converted into ventilation system airflow. The observed feature data of the constructed training sample set are normalized and used as input to the classifier for learning and training, and the binary vector of fault labels corresponding to multifault locations is used as the output of the classifier to train an RVMFL diagnostic classifier for mine ventilation. Based on the changes in air velocity at monitoring points when a ventilation system in normal production has a resistance variable multifault, the trained multifault location diagnostic classifier can quickly diagnose the locations of faults.

## Supervised machine learning model for RVMFL diagnosis in ventilation systems

### DT-based RVMFL diagnostic model

The DT algorithm supports multilabel classification problems. The process of RVMFL diagnosis is equivalent to constructing a fault label classification tree, which is based on the principle of learning multifault samples and inferring simple fault decision rules from data features to predict fault location target values^23–25^. When a wind volume vector *x*_*i*_ ∈ ***R***^*n*^, *i* = 1,…,*l* and its corresponding fault location label vector *y* ∈ **R**^*l*^ after a resistance variable multifault occur, the classification tree recursively divides the feature space so that samples with the same fault label are grouped. For each feature segmentation point, let $\Phi_{m}$ denote *n*_*m*_ multifault sample data at node *m*. For a candidate segmentation *θ* = (*j*, *t*_*m*_) comprising a fault feature *j* and a domain value *t*_*m*_, segment the data into subsets $\Phi_{m}^{a} \left( \theta \right)$ and $\Phi_{m}^{b} \left( \theta \right)$. The segmentation rules are as follows:

1 $$ \left\{ \begin{gathered} \Phi_{m}^{a} \left( \theta \right) = \left\{ {\left( {x,y} \right)|x_{j} \le t_{m} } \right\}, \hfill \\ \Phi_{m}^{b} \left( \theta \right) = \Phi_{m} - \Phi_{m}^{a} \left( \theta \right). \hfill \\ \end{gathered} \right. $$

The impurity function is used to calculate the number of impurities for one candidate segmentation of node *m*. The Gini index is chosen as the metric function for impurity and is calculated as follows:

2 $$ G\left( {\Phi_{m} ,\theta } \right) = \frac{{\left| {\Phi_{m}^{a} } \right|}}{{n_{m} }}Gini\left( {\Phi_{m}^{a} \left( \theta \right)} \right) + \frac{{\left| {\Phi_{m}^{b} } \right|}}{{n_{m} }}Gini\left( {\Phi_{m}^{b} \left( \theta \right)} \right), $$

3 $$ Gini\left( {\Phi_{m} } \right) = \sum\limits_{k} {p_{mk} \left( {1 - p_{mk} } \right)} , $$

where *p*_*mk*_ is the proportion of data labeled *k* at node *m*.

The parameters that minimize impurities are as follows:

4 $$ \theta^{*} = \arg \min_{\theta } G\left( {\Phi_{m} ,\theta } \right). $$

The recursion of subsets $\Phi_{m}^{a} \left( {\theta^{*} } \right)$ and $\Phi_{m}^{b} \left( {\theta^{*} } \right)$ until the maximum permissible depth is reached ends, which in turn generates a classification tree for the RVMFL diagnosis.

### MLP-based diagnostic model for RVMFL

MLP is a supervised learning algorithm that is a feed-forward network and supports multilabel classification problem solving^26–28^. Given a resistance variable multifault feature set and its corresponding fault labels, it can learn to obtain a nonlinear function approximator for multifault location diagnosis. Between the input and output layers of the multifault location diagnosis MLP model, there can be *l* (*l* ≥ 1) implicit layers with the following information-processing mechanism:

5 $$ O_{j}^{(l)} = g\left( {\sum\limits_{i = 1}^{{N_{l - 1} }} {w_{{_{ij} }}^{\left( l \right)} O_{i}^{l - 1} + b_{j}^{l} } } \right), $$

where $O_{j}^{(l)}$ is the output of the *j*th neuron in layer *l*, $O_{i}^{l - 1}$ is the output of the *i*th neuron in layer *l*-1, $w_{ij}^{\left( l \right)}$ is the connection weight of the *i*th neuron in layer *l*-1, and the *j*th neuron in layer *l*, $b_{j}^{l}$ is the bias of the *j*th neuron in layer* l*, and g(∙) is the activation function using the hyperbolic tangent as the activation function:

6 $$ g\left( z \right) = \frac{{e^{z} - e^{ - z} }}{{e^{z} + e^{ - z} }}. $$

The stochastic gradient descent algorithm was chosen and used to train this multifault location diagnosis perceptron network using the gradient of the loss function to update the weights w:

7 $$ w \leftarrow w - \eta \left( {\alpha \frac{\partial R\left( w \right)}{{\partial w}} + \frac{\partial Loss}{{\partial w}}} \right), $$

where *η* is the learning rate of the control step in the parameter space search.

The average cross-entropy is used as the loss function for the RVMFL diagnosis model, whose expression in the binary case takes the following form:

8 $$ Loss\left( {\hat{y},y,w} \right) = - \frac{1}{n}\sum\limits_{i = 0}^{n} {\left( {y_{i} \ln \hat{y}_{i} + \left( {1 - y_{i} } \right)\ln \left( {1 - \hat{y}_{i} } \right)} \right)} + \frac{\alpha }{2n}\left\| w \right\|_{2}^{2} , $$

where $\hat{y}$ is the predicted fault location, *y* is the actual fault location, *n* is the number of samples, $\alpha \left\| w \right\|_{2}^{2}$ is the L2 regularization term of the penalized complex model, and *α* > 0 is a nonnegative hyperparameter controlling the magnitude of the penalty.

Starting with initial random weights, MLP minimizes the loss function by iteratively updating these weights. After calculating the loss, backpropagation propagates it from the output layer to the previous layer, updating the value of each weight parameter to reduce the loss.

### Rank-SVM-based RVMFL diagnostic model

Rank-SVM is a ranking-based multilabel classification algorithm that uses a maximization interval strategy and introduces a kernel trick to address nonlinear classification problems^29–31^. Let the Rank-SVM multifault learning system comprise *m* linear classifiers ${\varvec{S}} = \left\{ {\left. {\left( {\omega_{j} ,b_{j} } \right)} \right|1 \le j \le m} \right\}$, where *ω*_*j*_ is the weight vector corresponding to the *j*th class of faults and *b*_*j*_ is the bias corresponding to the *j*th class of faults. For a given multifault training sample set ${\varvec{T}} = \left\{ {\left. {\left( {x_{i} ,Y_{i} } \right)} \right|1 \le i \le n} \right\}$, the multifault learning system produces classification intervals for the fault samples (*x*_*i*_,* Y*_*i*_), which can be expressed as follows:

9 $$ \mathop {\min }\limits_{{\left( {y_{i} ,y_{k} } \right) \in {\text{Y}}_{i} \times {\bar{\text{Y}}}_{i} }} \frac{{\left\langle {\omega_{j} - \omega_{k} ,x_{i} } \right\rangle + b_{j} - b_{k} }}{{\left\| {\omega_{j} - \omega_{k} } \right\|}}. $$

Equation (9) represents the distance of the fault samples to the classification hyperplane under each relevant–irrelevant marker pairing. By expanding the entire multifault training sample set ***T***, the classification interval of the learning system is given by

10 $$ \mathop {\min }\limits_{{\left( {x_{i} ,{\text{Y}}_{i} } \right) \in {\varvec{T}}}} \mathop {\min }\limits_{{\left( {y_{i} ,y_{k} } \right) \in {\text{Y}}_{i} \times {\bar{\text{Y}}}_{i} }} \frac{{\left\langle {\omega_{j} - \omega_{k} ,x_{i} } \right\rangle + b_{j} - b_{k} }}{{\left\| {\omega_{j} - \omega_{k} } \right\|}}. $$

The training sample set classification interval is considered positive, and the parameters of the linear classifier ***S*** are scaled. Then, the optimization problem of maximizing the training set classification interval can be expressed as follows:

11 $$ \begin{aligned} & \mathop {\max }\limits_{S} \mathop {\min }\limits_{{\left( {x_{i} ,Y_{i} } \right) \in T,\left( {y_{i} ,y_{k} } \right) \in Y_{i} \times \bar{Y}_{i} }} \frac{1}{{\left\| {\omega_{j} - \omega_{k} } \right\|^{2} }}, \\ & s.t.\;\left\langle {\omega_{j} - \omega_{k} ,x_{i} } \right\rangle + b_{j} - b_{k} \ge 1,\;\left( {1 \le i \le n,\left( {y_{j} ,y_{k} } \right) \in {\text{Y}}_{i} \times \bar{{\text{Y}}}_{i} } \right). \\ \end{aligned} $$

Let the training sample be sufficiently adequate, i.e., for all category markers* y*_*i*_ and *y*_*k*_; there exists $\left( {x,{\text{Y}}} \right) \in {\varvec{T}}$ such that $\left( {y_{j} ,y_{k} } \right) \in {\text{Y}} \times {\bar{\text{Y}}}$. Equation (11) can be transformed as follows:

12 $$ \begin{aligned} & \mathop {\min }\limits_{{\varvec{S}}} \mathop {\max }\limits_{1 \le j < k \le m} \left\| {\omega_{j} - \omega_{k} } \right\|^{2} , \\ & s.t.\;\left\langle {\omega_{j} - \omega_{k} ,x_{i} } \right\rangle + b_{j} - b_{k} \ge 1,\;\left( {1 \le i \le n,\left( {y_{j} ,y_{k} } \right) \in {\text{Y}}_{i} \times {\bar{\text{Y}}}_{i} } \right). \\ \end{aligned} $$

By approximating the max operator with a summation operator and introducing slack variables and using Ranking Loss as the loss, the optimization problem is transformed into

13 $$ \begin{aligned} & \min \sum\limits_{j = 1}^{m} {\left\| {\omega_{j} } \right\|^{2} + C\sum\limits_{i = 1}^{n} {\frac{1}{{\left| {Y_{i} } \right|\left| {\bar{Y}_{i} } \right|}}} } \sum\limits_{{\left( {y_{i} ,y_{k} } \right) \in Y_{i} \times \bar{Y}_{i} }} {\xi_{ijk} } , \\ & s.t.\;\left\langle {\omega_{j} - \omega_{k} ,x_{i} } \right\rangle + b_{j} - b_{k} \ge 1 - \xi_{ijk} , \\ & \;\;\;\;\;\xi_{ijk} > 0\;\;\;\left( {1 \le i \le n,\left( {y_{j} ,y_{k} } \right) \in {\text{Y}}_{i} \times \bar{{\text{Y}}}_{i} } \right), \\ \end{aligned} $$

where *C* is the equilibrium coefficient and $\xi_{ijk}$ is the relaxation variable, $\xi_{ijk}$ ≥ 0.

### Model evaluation metrics

To compare and evaluate the diagnostic performance of DT, MLP, and Rank-SVM-based RVMFL diagnosis models for mine ventilation systems, the evaluation metrics were selected considering the existing literature on multilabel classification problems ^32–35^. The five metrics of hamming loss, ranking loss, coverage, average accuracy, and one-error are the most commonly used and most widely applied metrics. These five metrics were selected to evaluate the RVMFL diagnosis models for mine ventilation, as shown in Table 1. $y \in \left\{ {0,1} \right\}^{N \times M}$ in Table 1 denotes the binary label matrix corresponding to the real labels at multifault locations, and $\hat{f} \in {\varvec{R}}^{N \times M}$ denotes the score for each fault label.

**Table 1:** Performance evaluation metrics of the RVMFL diagnosis model.

Evaluation metrics	Calculation formula	Evaluation criteria
Hamming loss	$HL\left( {y,\hat{y}} \right) = \frac{1}{M}\sum\limits_{i = 0}^{M - 1} {1\left( {\hat{y}_{i} \ne y_{i} } \right)}$	The smaller the value, the better the performance
Ranking loss	$RL\left( {y,\hat{f}} \right) = \frac{1}{N}\sum\limits_{i = 0}^{N - 1} {\frac{1}{{\left\| {y_{i} } \right\|_{0} \left( {M - \left\| {y_{i} } \right\|_{0} } \right)}}} \left| {\left\{ {\left( {k,l} \right):\hat{f}_{ik} \le \hat{f}_{il} ,y_{ik} = 1,y_{il} = 0} \right\}} \right|$	The smaller the value, the better the performance
Coverage	$Cov\left( {y,\hat{f}} \right) = \frac{1}{N}\sum\limits_{i = 0}^{N - 1} {\mathop {\max }\limits_{j:yij = 1} } \;\text{rank}_{ij} - 1$, ${\text{rank}}_{ij} = \left| {\left\{ {k:\hat{f}_{ik} \ge \hat{f}_{ij} } \right\}} \right|$	The smaller the value, the better the performance
Average precision	$AP\left( {y,\hat{f}} \right) = \frac{1}{N}\sum\limits_{i = 0}^{N - 1} {\frac{1}{{\left\| {y_{i} } \right\|_{0} }}\sum\limits_{j:yij = 1} {\frac{{\left| {{\mathbf{L}}_{ij} } \right|}}{{{\text{rank}}_{ij} }}} }$, ${\mathbf{L}}_{ij} = \left| {\left\{ {k:y_{ik} = 1,\hat{f}_{ik} \ge \hat{f}_{ij} } \right\}} \right|$	The higher the value, the better the performance
One-error	$OE\left( {y,\hat{f}} \right) = \frac{1}{N}\sum\limits_{i = 0}^{{N{ - 1}}} {\left\{ {\left[ {\arg \max \hat{f}} \right] \notin y_{i} } \right\}}$	The smaller the value, the better the performance

## The RVMFL diagnosis experiment for ventilation system

### Experimental methods

**Figure 3:**
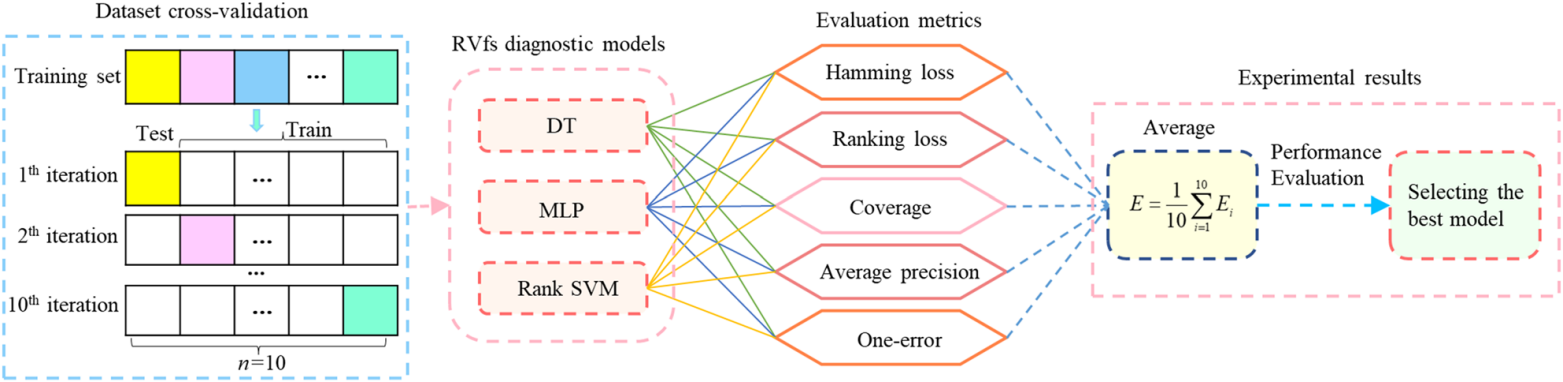
The experimental study process for RVMFL diagnosis.

To verify the feasibility and model reliability of the DT, MLP, and Rank-SVM-based RVMFL diagnosis method for ventilation systems, as well as to compare and analyze the diagnostic performance of these three models and find the optimal model, experiments were conducted using a 10-branch simple T-shaped angle-joint network and a 54-branch network with a multifault location diagnosis experimental system. The experimental study process is shown in Fig. 3. Using five evaluation metrics, tenfold cross-validation was used to train and validate the multifault location diagnosis sample set. The cross-validation results were statistically averaged. Real multifault diagnosis experiments were conducted using a ventilation system to verify the reliability of the model.

## T-shaped angle-joint ventilation network fault diagnosis experiment

### T-shaped angle-joint ventilation network

**Figure 4:**
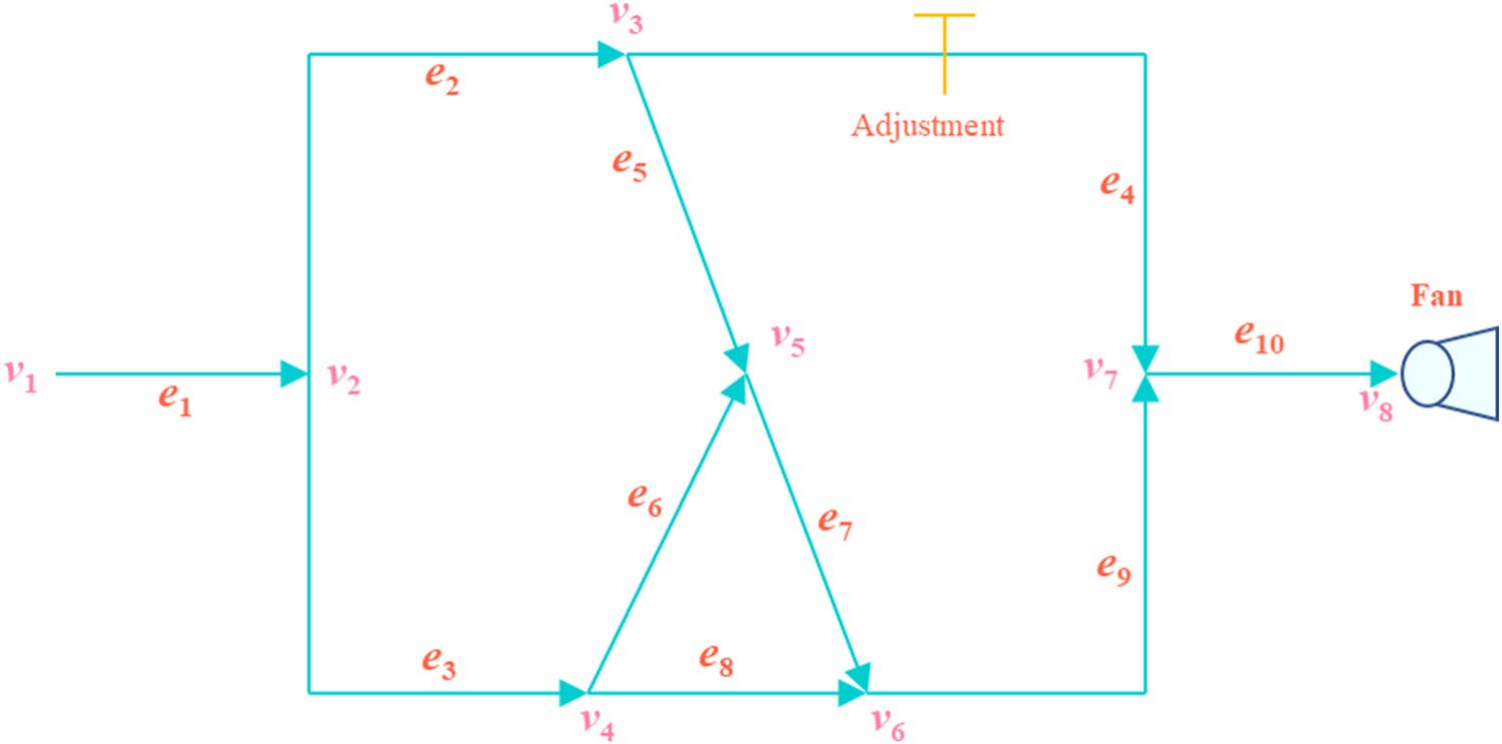
T-shaped angle-joint ventilation network.

The topology of the T-shaped angle-joint ventilation network, the wind resistance coefficient and the fan characteristic curves in literature 6 are used as references for the ventilation system RVMFL diagnosis study. Figure 4 shows a T-shaped angle-joint ventilation network with 8 nodes and 10 branches, with adjustment at branch *e*_4_ and a ventilation fan at branch *e*_8_. The characteristic curve of the ventilation fan is given as follows:

14 $$ H(q) = 1035.92 + 51.73q - 0.43q^{2} . $$

The branching parameters of the T-shaped angle-joint ventilation network are presented in Table 2.

**Table 2:** The branching parameters of the T-shaped angle-joint ventilation network.

Branch	Nodes	r/(N s^2^ m^−8^)	q/(m^3^ s^−1^)	Branch	Nodes	r/(N s^2^ m^−8^)	q/(m^3^ s^−1^)
e_1_	(v_1_,v_2_)	0.055	89.798	e_6_	(v_4_,v_5_)	0.110	15.023
e_2_	(v_2_,v_3_)	0.175	34.825	e_7_	(v_5_,v_6_)	0.190	34.834
e_3_	(v_2_,v_4_)	0.075	54.973	e_8_	(v_4_,v_6_)	0.160	39.951
e_4_	(v_3_,v_7_)	4.050	15.014	e_9_	(v_6_,v_7_)	0.115	74.784
e_5_	(v_3_,v_5_)	0.100	19.811	e_10_	(v_7_,v_8_)	0.080	89.798

### Construction of a multifault sample set for the T-shaped angle-joint ventilation network

Based on the branch information of the ventilation network during normal production periods, the mine ventilation simulation system (MVSS)^36^ is used to simulate the resistance-increasing faults of the general branches, except for the inlet and outlet branches, and the resistance-decreasing faults of the dampers. Assuming that the two branches in the ventilation system fail simultaneously and that the faulted air resistance values are generated using a random method, the steps for generating the resistive multifault sample set are as follows:

1. Keeping the topology of the ventilation network and the operating characteristics of the ventilator unchanged when any two branches of the ventilation system *e*_*i*_ and *e*_*j*_ have faulted simultaneously, with resistance variables Δ*r*_*i*_ and Δ*r*_*j*_, the wind resistances of the faulty branch become $r^{\prime}_{i} = r_{i} \pm \Delta r_{i}$ and $r^{\prime}_{j} = r_{j} \pm \Delta r_{j}$, respectively.
2. Based on the wind resistance vector $\user2{R^{\prime}}_{\left( i \right)} = \left( {r_{1} ,r_{2} , \cdots ,r^{\prime}_{i} , \cdots ,r^{\prime}_{j} , \cdots r_{n} } \right)$ of the ventilation network at the time of branch *e*_*i*_ and *e*_*j*_ failures, the ventilation network after the fault is solved once it generates new sample data $\user2{Q^{\prime}}_{\left( i \right)} = \left( {q^{\prime}_{1} ,q^{\prime}_{2} , \cdots ,q^{\prime}_{n} } \right)$ for the air volume.
3. Construct a multifault sample data space and record the branch numbers *e*_*i*_ and *e*_*j*_ where the fault occurred and the ventilation system branch air volume $\user2{Q^{\prime}}_{\left( i \right)}$ as a sample in the fault sample data space.
4. Repeat steps (1)–(3) so that the *e*_*i*_ and *e*_*j*_ branches occur many times, and the number of fault variables differs with each occurrence, resulting in generating the resistance multifault samples on the *e*_*i*_ and *e*_*j*_ branches. Based on the above rules, other branches of the ventilation network generate multifault samples of the corresponding branches, forming the ventilation network resistance variable multifault sample set ***T***.

Following the above method, a total of 600 sets of resistance variable multifault samples were generated for the T-shaped angle-joint ventilation network, as shown in Table 3. The observed features of the multifault sample set data were normalized before being input to the multifault location diagnostic classifier.

**Table 3:** The sample set of multifaults for the T-shaped angle-joint ventilation network.

Number	$q^{\prime}_{1}$	$q^{\prime}_{2}$	$q^{\prime}_{3}$	$q^{\prime}_{4}$	$q^{\prime}_{5}$	$q^{\prime}_{6}$	$q^{\prime}_{7}$	$q^{\prime}_{8}$	$q^{\prime}_{9}$	$q^{\prime}_{10}$	e_i_, e_j_
1	88.34	36.70	51.64	14.86	21.84	12.63	34.48	39.00	73.48	88.34	e_2_, e_3_
2	86.97	37.58	49.40	14.69	22.89	11.16	34.05	38.24	72.29	86.97	e_2_, e_3_
3	88.49	33.51	54.98	14.76	18.74	15.51	34.26	39.47	73.72	88.49	e_2_, e_3_
…	…	…	…	…	…	…	…	…	…	…	…
598	35.89	18.32	17.57	28.49	− 10.17	15.16	4.99	2.41	7.4	35.89	e_8_, e_9_
599	35.07	18.1	16.97	28.62	− 10.52	14.98	4.45	1.99	6.44	35.07	e_8_, e_9_
600	34.50	17.94	16.55	28.71	− 10.77	14.83	4.06	1.73	5.78	34.5	e_8_, e_9_

### Parameter setting

The reasonableness of the hyperparameter settings determines the predictive performance of the model's multifault location diagnosis machine learning model. In this study, the cross-validation grid search method is used to determine the hyperparameters of the model. The hyperparameter settings of the T-shaped angle-joint ventilation network multifault location diagnosis machine learning model are shown in Table 4.

**Table 4:** Super parameter settings for the T-shaped angle-joint ventilation network multifault location diagnosis model.

Models	Parameters					
DT	Max depth	Criterion	Splitter	Min samples split	Min samples leaf	
	122	Geni	best	2	1	
MLP	Hidden layer	Hidden layer sizes	Max iter	Activation function	Solver	alpha
	1	52	5000	tanh	lbfgs	0.0001
Rank-SVM	Type	Cost	Max iter	lambda		
	Linear	1	50	0.000001		

### Experimental results

**Figure 5:**
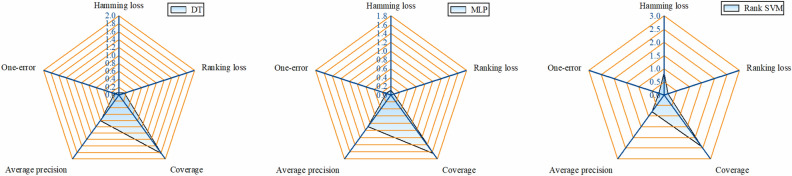
Cross-validation results for RVMFL diagnosis of the T-shaped angle-joint ventilation network.

A sample set containing 600 sets of multifaults was cross-validated. The results are shown in Fig. 5. As shown in Fig. 5, the DT-based RVMFL diagnosis model has a hamming loss of 0.061, which is the smallest among the three models. The MLP-based RVMFL diagnosis model has a ranking loss, coverage, and one-error of 0.067, 1.643, and 0.117, respectively, which are the smallest among the three models, and its average accuracy of 0.889 is the highest among the three models, and it has a hamming loss of 0.106. The Rank-SVM-based RVMFL diagnostic model has the worst predictive metrics of all three models. It can be observed that all three algorithms, DT, MLP, and Rank-SVM, can be used to diagnose multifault locations in mine ventilation, and the method is feasible. The MLP model exhibits the best prediction performance, best generalization ability, and highest prediction accuracy for the multifault sample dataset of the T-shaped angle-joint ventilation network. Furthermore, the DT model is slightly lower than the MLP in all metrics except for hamming loss but higher than the Rank-SVM, i.e., the predictive performance and generalization ability of the DT model is slightly lower than that of the MLP and much higher than that of the Rank-SVM.

## Experimental ventilation system multifault diagnosis experiment

### Resistance variable multifault diagnostic experimental system

**Figure 6:**
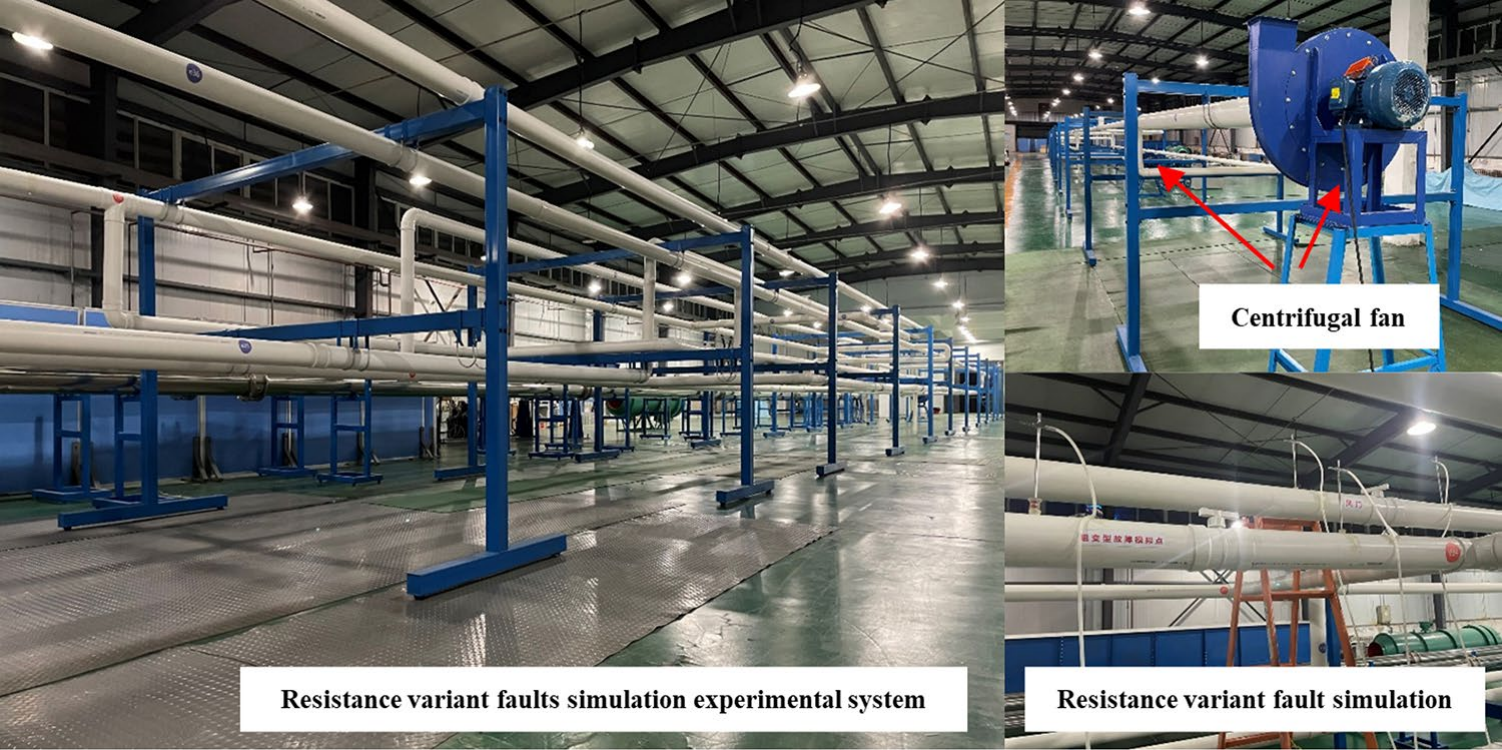
RVMFL diagnosis experimental system.

The experimental ventilation system was built using unplasticized polyvinyl chloride (UPVC) pipes with diameters of 200 mm, 160 mm, and 110 mm. The total length of the experimental system piping is 353 m. There are 14 structures in the experimental system, of which seven are completely closed, one is the inlet air branch adjustment, and the remaining six are fault-simulated adjustment valves. The system can simulate different types and amounts of faults, and it can better simulate situations in which multiple faults occur in the ventilation system. The seven closed dampers position can be carried out to simulate the descending resistance fault experiment, and the six regulating valve position can be carried out to simulate the increasing resistance fault experiment. The experimental system is shown in Fig. 6. The system comprises two inlet and two outlet air pipelines. The inlet air of the system comprised UPVC pipes with a diameter of 160 mm, the outlet air of the system comprised UPVC pipes with a diameter of 200 mm, and the rest of the main part comprised UPVC pipes with a diameter of 110 mm. The data collection system of the experiment includes a TSI 9565P ventilation parameter tester and Pitot tube. The accuracy of wind velocity testing using a pitot tube is ± 1.5% at 2000 ft/min. The principle of the experiment is to test the velocity pressure to get the velocity at the center of the pipeline section and convert it into the average air velocity. The velocity of the wind was tested by using TSI to monitor continuously for two minutes and taking the average value. Because the pipeline used in the experiment is a relatively smooth industrial UPVC pipe, according to Moody diagrams and actual ventilation resistance tests, the calculated flow indices of the ventilation resistance of these three pipelines are 1.834, 1.849, and 1.812, respectively. Two centrifugal fans are installed at the end of each of the two return air pipes, and airflow control valves are arranged inside the system as airflow control facilities.

**Figure 7:**
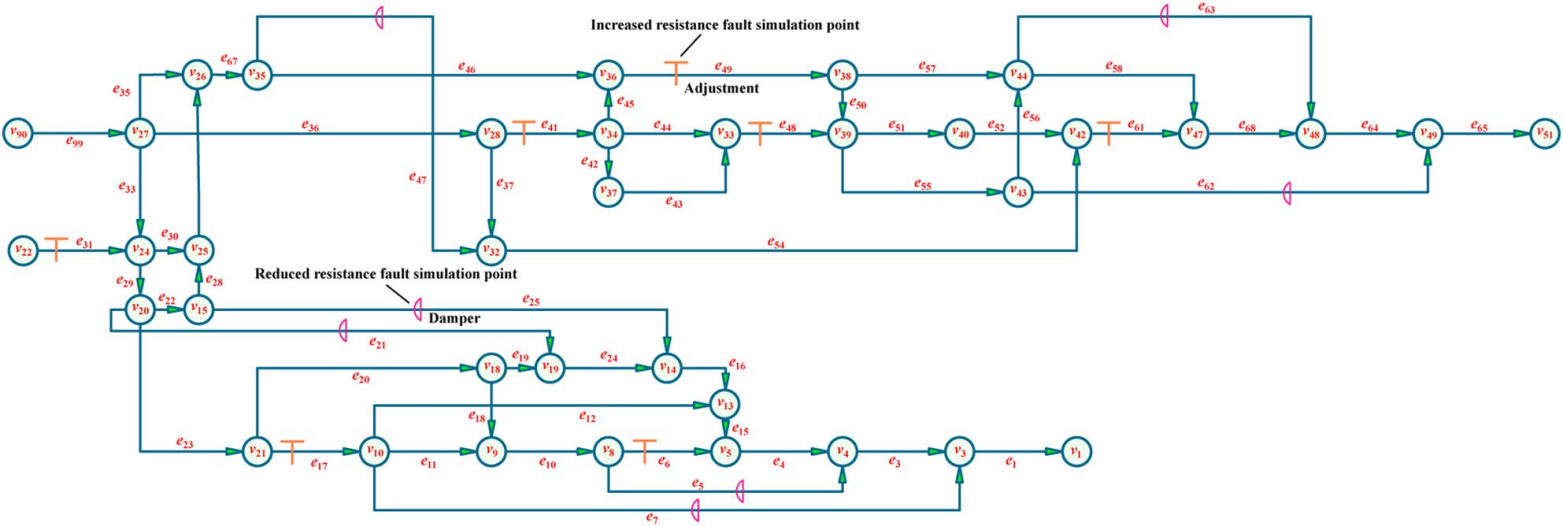
Experimental system topology.

The topology of the experimental system is illustrated in Fig. 7. The model 9–26-4A 5.5KW centrifugal fan is installed on branch *e*_1_, and the model 9–19-5A 7.5KW centrifugal fan is installed on branch *e*_65_. The operating frequency of both fans is 50 Hz. The plate resistance method is used to increase the resistance of the system, and the air volume and pressure of the ventilation fan under different system conditions are tested by using a differential pressure meter and pitot tube, and the characteristic curve equation of the fan is obtained by the method of data fitting. By testing, the characteristic curves of these fans are h (*q*) = 3156.2 + 1323.6 *q*—1838.7 *q*^2^ and *h* (*q*) = 4266.8 + 4515.3 *q*—4406.1 *q*^2^, respectively.

### Multifault sample and parameter setting for the experimental ventilation system

Based on the construction method of the T-shaped angle-joint ventilation network resistance variable multifault sample set, 500 sets of experimental ventilation network multifault sample sets were generated, as shown in Table 5.

**Table 5:** Experimental ventilation network multifault sample set.

Number	$q^{\prime}_{5}$	$q^{\prime}_{7}$	$q^{\prime}_{12}$	$q^{\prime}_{16}$	$q^{\prime}_{20}$	$q^{\prime}_{21}$	$q^{\prime}_{22}$	$q^{\prime}_{25}$	…	$q^{\prime}_{63}$	e_i_, e_j_
1	0.09170	0.13742	0.09475	0.09069	0.10337	0.06194	-0.00140	0.03135	…	0.01408	e_5_, e_7_
2	0.10827	0.13956	0.09389	0.09032	0.10487	0.06284	-0.00245	0.03179	…	0.01407	e_5_, e_7_
3	0.11640	0.14016	0.09364	0.09023	0.10552	0.06323	-0.00290	0.03199	…	0.01406	e_5_, e_7_
…	…	…	…	…	…	…	…	…	…	…	…
498	0.00344	0.06549	0.10942	0.09618	0.08473	0.05050	0.02247	0.02545	…	0.01233	e_61_, e_63_
499	0.00344	0.06547	0.10937	0.09613	0.08469	0.05048	0.02294	0.02542	…	0.01222	e_61_, e_63_
500	0.00344	0.06546	0.10936	0.09612	0.08468	0.05047	0.02307	0.02541	…	0.01219	e_61_, e_63_

A cross-validation grid search was adopted to determine the hyperparameters of the experimental ventilation network multifault location diagnosis model, as shown in Table 6.

**Table 6:** Experimental ventilation network multifault location diagnosis model hyperparameter setting.

Models	Parameters					
DT	Max depth	Criterion	Splitter	Min samples split	Min samples leaf	
	125	Geni	best	2	1	
MLP	Hidden layer	Hidden layer sizes	Max iter	Activation function	Solver	alpha
	1	36	5000	tanh	lbfgs	0.0001
Rank-SVM	Type	Cost	Max iter	lambda		
	Linear	1	50	0.000001		

### Experimental results

**Figure 8:**
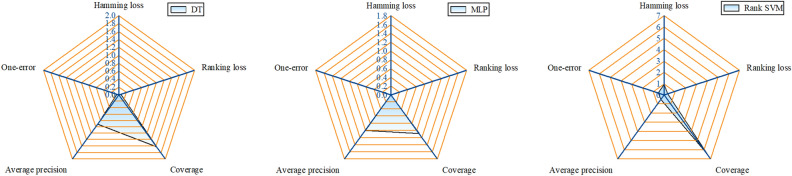
Cross-validation results of the experimental system for RVMFL diagnosis.

Cross-validation was performed on a 500-group multifault sample set, and the results of the cross-validation are shown in Fig. 8. As illustrated in the figure, the MLP-based multifault location diagnosis model exhibits the smallest hamming loss, ranking loss, coverage, and one-error among the three models, which are 0.012, 0.004, 1.086, and 0.002, respectively, with the highest average accuracy of 0.992. The DT model’s hamming loss, ranking loss, coverage, one-error, and precision were 0.014, 0.05, 1.63, 0.056, and 0.916, respectively, and they exhibited slightly lower metrics than those of the MLP model. The Rank-SVM model has the worst metrics among the three models. It can be observed that the predictive performance and generalization ability of the MLP-based RVMFL diagnosis model are better than those of the DT and Rank-SVM models.

In the experimental system, increasing and decreasing the resistance fault simulation point improve the reliability of the multifault location diagnostic model; thus, the ventilation system experiences resistance variable multifaults. Owing to the poor predictive performance and generalization ability of the Rank-SVM model for multifault sample sets, it is not considered here; only the reliability of the MLP and DT models is considered. A fully open regulating valve preset at the simulation point of a resistance-increasing fault in the experimental ventilation system is used to create a resistance-increasing fault, and the dampers in the original system are used to create a resistance-reducing fault. A total of 9 sets of multifaults were created and tested for the remaining branch airflow in each state. After each multifault diagnosis was performed, the system was restored to the original ventilation system state to ensure consistency. A sample of the experimental ventilation system multifault example tests is shown in Table 7.

**Table 7:** Experimental ventilation system with a multifault instance test sample.

Number	$q^{\prime}_{5}$	$q^{\prime}_{7}$	$q^{\prime}_{12}$	$q^{\prime}_{16}$	$q^{\prime}_{20}$	$q^{\prime}_{21}$	$q^{\prime}_{22}$	$q^{\prime}_{25}$	…	$q^{\prime}_{63}$	e_i_, e_j_
1	0.09269	0.14028	0.09426	0.09057	0.10384	0.06224	− 0.00177	0.03150	…	0.01407	e_5_, e_7_
2	0.00354	0.06723	0.10921	0.10307	0.07496	0.06243	0.02925	0.05186	…	0.01417	e_21_, e_25_
3	0.00311	0.05870	0.08679	0.10068	0.14650	0.07325	0.03336	0.03635	…	0.01342	e_17_, e_41_
4	0.00349	0.06651	0.11108	0.09773	0.08594	0.05122	0.00363	0.02623	…	0.01427	e_49_, e_61_
5	0.00348	0.06626	0.11067	0.09732	0.08566	0.05106	0.01630	0.02593	…	0.01318	e_47_, e_48_
6	0.00345	0.06558	0.10956	0.09631	0.08483	0.05056	0.02104	0.02553	…	0.01257	e_62_, e_61_
7	0.00344	0.06539	0.10928	0.09596	0.08469	0.05047	0.03256	0.02502	…	0.18318	e_47_, e_63_
8	0.00342	0.06518	0.10895	0.09564	0.08447	0.05034	0.03580	0.02479	…	0.01194	e_47_, e_62_
9	0.00337	0.05999	0.11029	0.11489	0.13920	0.07010	0.02597	0.03521	…	0.01423	e_6_, e_17_

The reliability of the MLP and DT models was verified using the multifault sample set in Table 5 as the training set and the multifault example test sample of the experimental ventilation system in Table 7 as the test set. The MLP multifault location diagnosis model was used to diagnose all 9 groups of multifault locations with an accuracy rate of 100%. With a diagnostic accuracy rate of 44.44%, the DT multifault location diagnosis model was used to accurately diagnose two fault locations in 4 groups, of which one fault location was diagnosed accurately in 4 groups and one group was not diagnosed accurately in both locations. Using the existing model of ML-KNN to diagnose the multifault locations of the experimental system, the diagnostic accuracy is 88.89% when *k* = 2 and 100% when *k* = 3. The diagnostic accuracy of the MLP model is comparable to that of the ML-KNN model.

## Conclusion

This study investigated the concurrent diagnosis of resistance faults occurring at multiple locations in a mine ventilation system, proposed three supervised machine learning diagnosis models for RVMFL diagnosis, and validated the reliability and effectiveness of the models and methods using a 10-branch T-shaped angle-joint ventilation network and a 54-branch experimental ventilation network. We obtained the following main conclusions:

1. According to the cross-validation results, the DT, MLP, and Rank-SVM supervised machine learning methods are feasible for the multifault location diagnosis of mine ventilation systems based on air volume characteristics. The diagnostic performances of both the MLP and DT models are better than that of the Rank-SVM model, and the MLP model performs the best.
2. In the diagnosis of the experimental ventilation system resistance variable multifault instance, the diagnostic accuracy of the MLP model is 100%, while the diagnostic accuracy of the DT model is 44.44%, further indicating that the generalization ability of the MLP model is better than that of the DT model. The high diagnostic accuracy and reliability satisfy the engineering requirements and can be used as a method of RVMFL diagnosis in engineering practice and application.
3. The successful practice of RVMFL diagnosis of experimental ventilation systems shows that the resistance variable multifault experimental verification system for mines established in this study can serve as a verification platform for intelligent fault diagnosis of mine ventilation systems, effectively solving the problem that industrial tests cannot be conducted in the field and providing strong support for the construction of intelligent ventilation systems for mines.

This study focused on the diagnosis of multifault locations in mine ventilation systems, and further study is needed to diagnose the magnitude of faults, i.e., the volume of faults, in ventilation systems where multifault locations occur. In this study, only the air volume was used as a single feature as an input to the model, and a higher accuracy might be obtained if factors such as the differential pressure of the structure or pressure energy of the nodes were considered. Rank-SVM has the worst diagnostic performance among the three models. The factors affecting the performance of the Rank-SVM model are the setting of the penalty factor, the selection of the kernel, and the sample size and quality. At present, the kernel selected in this paper is a linear kernel and the penalty factor set in this paper may lead to the degradation of the diagnostic performance of the model, and the subsequent research should focus on the factors affecting the performance of the model in order to find the best model setting so as to improve the diagnostic performance of the model.

## Data Availability

All relevant data are within the paper.
